# Differential effects of contextual congruency on recognition and retrieval of perceptual details

**DOI:** 10.3758/s13421-025-01848-0

**Published:** 2026-02-03

**Authors:** Rebeca Suárez, Bruno Lara, Alejandra Ciria

**Affiliations:** 1 Facultad de Psicología, Universidad Nacional Autónoma de México, Ciudad de México, México; 2 Centro de Investigación en Ciencias, Universidad Autónoma del Estado de Morelos, Cuernavaca, Morelos México; 3 Instituto de Investigaciones en Matemáticas Aplicadas y en Sistemas, Universidad Nacional Autónoma de México, Ciudad de México, México

**Keywords:** Contextual congruency, Memory schemas, Prediction, Recognition, Retrieval

## Abstract

Prediction errors arising from contextual violations play a fundamental role in learning and memory, yet their effects remain controversial. While some research suggests prediction errors enhance memory for incongruent information, other evidence shows that schema-congruent events are better remembered. This study investigates how *contextual congruency* during encoding affects both recognition memory and retrieval of fine-grained perceptual details. Using object–scene pairings, we examined whether predictions based on memory schemas differentially influence the encoding of congruent versus incongruent information. The Mnemonic Similarity Task (MST) was adapted to incorporate naturalistic scene contexts. Participants viewed indoor scenes with contextually congruent or incongruent objects during encoding, then classified single objects as ”old” (previously encountered at the encoding phase), ”similar” (objects perceptually similar to previously encountered), or ”new”. Memory was assessed using the Corrected Recognition score (REC) for recognition accuracy, the Lure Discrimination Index (LDI) for fine-grained perceptual detail retrieval and discrimination, and the Rate of Correct Scores (RCS) for processing efficiency. Contextually congruent objects yielded significantly higher recognition accuracy and processing efficiency compared to incongruent objects. However, no congruency advantage was found for the retrieval of fine-grained perceptual details, with equivalent performance across conditions. These findings suggest that predictions based on memory schemas enhance encoding and retrieval of general item information, facilitating recognition and reducing cognitive demands. In contrast, encoding of fine-grained perceptual details appears unaffected by *contextual congruency*. These differential effects between recognition and retrieval of perceptual details offer important insights into how predictions influence distinct aspects of memory encoding.

## Introduction

Imagine encountering a penguin when you are walking on a busy, noisy, familiar street. This unexpected event violates deeply ingrained contextual predictions and likely triggers a state of surprise, signaling that something does not match preexisting knowledge about the world. Such experiences are central to how we learn and adapt, yet their impact on memory remains unclear. Every day experiences with the world continuously shape our predictions about which objects are likely to appear together in a scene and where they will be located. When these predictions are not met (prediction violations), the resulting prediction error plays a key role in learning (Clark, 2013; Hohwy, 2013; Rescorla, 1972; van Kesteren, Ruiter, Fernández, & Henson, 2012; von Restorff, 1933), as it signals novel information that can be integrated into preexisting knowledge (Quent, Henson, & Greve, 2021). However, several controversies exist regarding the effects of prediction error on memory encoding and retrieval (Bein, Gasser, Amer, Maril, & Davachi, 2023). Some studies provide evidence that prediction error enhances memory for those events or elements that violate predictions (Bein, Plotkin, & Davachi, 2021; Greve, Cooper, Kaula, Anderson, & Henson, 2017; Kafkas & Montaldi, 2018; Prull, 2015; Quent, Greve, & Henson, 2022; von Restorff, 1933). In contrast, other research suggests that congruent inputs, those that align with predictions, facilitate and promote retrieval (Audrain & McAndrews, 2022; Bein et al., 2015; Nolden, Turan, Bein, Davachi, & Shing, 2025; Ortiz-Tudela, Milliken, Botta, LaPointe, & Lupiañez, 2017; Ortiz-Tudela et al., 2023; Sommer, Hennies, Lewis, & Alink, 2022; van Kesteren et al., 2012; Yousuf, Packard, Fuentemilla, & Bunzeck, 2021).

Given these inconsistencies, the central question guiding this work is whether the congruency of information with preexisting knowledge influences not only whether an event is remembered, but also how it is remembered, specifically, whether fine-grained perceptual details are encoded and can later be retrieved. Thus, this work aims to clarify whether *contextual congruency*, defined by the semantic likelihood of object–scene combinations, differentially affects both memory recognition and retrieval of fine-grained perceptual details, a less explored dimension of memory performance. This contribution is made possible by combining the use of naturalistic object–scene pairings with an experimental design that enables detecting performance differences in object recognition and retrieval of fine-grained perceptual details under congruent and incongruent conditions.

Numerous behavioral and neuroscientific studies have shown that context-driven predictions facilitate the perception of semantically congruent objects by enabling faster and more accurate identification (Bar, 2004; Brandman & Peelen, 2017; Davenport & Potter, 2004; Oliva & Torralba, 2007; Palmer, 1975; Torralba, 2003). From the perspective of predictive coding models of perception (Clark, 2013; De Lange, Heilbron, & Kok, 2018; Friston & Kiebel, 2009; Hohwy, 2013), these congruency effects reflect the brain’s use of prior knowledge to form probabilistic predictions about likely stimuli in a given scene. In the present study, a statistical definition of prediction is adopted, one that does not imply explicit predictions of future events, but rather reflects the brain’s ongoing probabilistic estimation of incoming sensory information based on learned regularities (De Lange et al., 2018). Under this framework, the brain continuously generates predictions about what is likely to occur in a given context, and deviations from these predictions produce measurable prediction errors even without explicit anticipation.

Most research on prediction error effects in episodic memory has relied on paradigms that require participants to learn item–item associations or undergo training phases prior to encoding, thereby ensuring that a specific representation is activated before the critical event occurs (Bein et al., 2021; Quent et al., 2022). Under these conditions, predictions are made explicitly, and violations of these predictions produce prediction error signals with measurable consequences on memory, as well as on other physiological measures (Brod, Greve, Jolles, Theobald, & Galeano-Keiner, 2022; Brod, Hasselhorn, & Bunge, 2018). While this approach is valuable and has shown that explicit predictions can enhance memory encoding, it does not preclude the possibility that predictions, and their associated errors, can also operate implicitly through activation of preexisting knowledge during perception and action.

Memory relies on past experiences to generate predictions that shape how new information is encoded and later retrieved (Bar, 2004; Bein et al., 2021; Brod, WerkleBergner, & Shing, 2013; Frank & Kafkas, 2021; Friston & Kiebel, 2009; Pearce & Bouton, 2001; Pupillo, Ortiz-Tudela, Bruckner, & Shing, 2023). These predictions help guide perception and attention toward relevant elements in familiar contexts. The stored knowledge from past experiences is organized into memory schemas, defined by Ghosh and Gilboa (2014) as flexible associative networks formed through repeated similar experiences, yet lacking in specific detail. Prior knowledge structured in schemas not only guides attention but also supports the encoding, consolidation, and long-term retention of new information (Alonso, van der Meij, Tse, & Genzel, 2020; Fernández & Morris, 2018).

Schemas play a central role in learning, since predictions are generated based on this prior knowledge (Alonso et al., 2020; Bartlett, 1995; Fernández & Morris, 2018). During the perception of scenes or stimuli, schemas are rapidly activated and function as predictive templates that direct attention and interpretation based on prior knowledge (Bein et al., 2023). Thus, the encoding of new information can either contribute to the formation of a new schema or refine, expand, and enhance an existing one. The *congruency effect* illustrates the role of prior knowledge in memory by showing that items are better remembered when presented in contexts compatible with existing semantic knowledge (Bein et al., 2015; Brod & Shing, 2022; Craik & Tulving, 1975; Schulman, 1974). This effect has been observed across various stimuli, modalities (Bein et al., 2015; Craik & Tulving, 1975; Pan, Zhang, Hu, & Zuo, 2022; Quent et al., 2022; Staresina, Gray, & Davachi, 2009; van Kesteren et al., 2013), encoding conditions (intentional and incidental), and memory tests, including recall and recognition (Bein et al., 2015; Craik & Tulving, 1975; Persaud, Macias, & Bonawitz, 2025; Schulman, 1974).

Beyond behavioral findings, event-related potential (ERP) evidence suggests that contextual violations in visual scenes elicit early and late neural responses (N300 and N400-like components) even when the object and scene are presented simultaneously, without the opportunity for prior expectation formation (Mudrik, Lamy, & Deouell, 2010). The N300 is a late visual event-related potential component that reflects intermediate-to-late stages of predictive hypothesis testing during visual processing, capturing the integration of perceptual input with prior knowledge and contextual predictions, and often precedes and co-occurs with the N400, which indexes access to multimodal semantic memory (Kumar, Federmeier, & Beck, 2021). In this regard, Kumar et al. (2021) found that the N300 amplitude was significantly larger for statistically irregular exemplars of contexts in comparison to prototypical exemplars of contexts, suggesting that this component reflects the integration of visual input with prior knowledge. Additionally, in the experiment of Guillaume, Baier, and Etienne (2020), scenes (e.g., a basketball game) with objects being handled were presented with either congruent (basketball) or incongruent (watermelon) objects, and a greater N400 amplitude was observed in the incongruent conditions compared to the congruent conditions. These findings indicate that congruency effects may reflect fast online matching processes between scene schemas and potential objects, supporting the idea that incongruent objects can elicit prediction-like violations during scene processing. Additionally, it is important to note that a greater neural response may reflect the additional processing needed to resolve a mismatch between the predicted input and the incoming stimulus. Still, this processing does not guarantee that this information will be integrated into existing memory schemas.

Consistent with this view, studies show that congruent object–scene pairs are better remembered than incongruent ones. In van Kesteren et al. (2013), participants memorized pairs of photographs representing objects and scenes, with an assessment of the congruence between objects and scenes. A memory test conducted one day later revealed that congruent object–scene pairs were better remembered than incongruent ones, a finding replicated in Ortiz-Tudela et al. (2017). Similarly, in an experiment using congruent object–scene pairs, memory was tested after both a short delay (10 min) and a long delay (3 days), consistently showing a memory advantage for congruent pairs (Audrain & McAndrews, 2022). Additionally, evidence suggests that semantic relationships promote memory integration, as observed when presenting pairs of images related to the same memory schema (Frank, Montaldi, Wittmann, & Talmi, 2018). Importantly, it has been observed that schema-congruent spatial locations enhance memory mainly when episodic memory is weak or unavailable, while strong recollection can override schema-based biases (Ramey, Henderson, & Yonelinas, 2022). In this regard, it is suggested that age-related increases in such schema-based biases are largely driven by recollection failures (Brod & Shing, 2022; Ramey, Yonelinas, & Henderson, 2024).

Although it is well accepted that semantic congruence benefits memory, a substantial body of research has also shown that events incongruent with the prevailing context can be well remembered (Brod et al., 2013; van Kesteren et al., 2012). Classic examples include studies by von Restorff (1933) using simple stimulus lists, where an odd item (oddball) that differs from its surrounding items is more likely to be remembered than those congruent with the list’s context. This finding, known as the von Restorff effect, is robust and widely used in memory and prediction research (MacLeod, 2020). Additionally, Greve et al. (2017) found that when items violated previously learned word–scene associations, associative memory between items and their background scene images improved. In this study, participants learned through repeated exposure that different scene categories were associated with words of positive or negative valence. This learned expectation was later violated by altering the word valence following a given scene, and memory for the word–scene association was stronger for words that violated previously learned contingencies. Similarly, Kafkas and Montaldi (2018) taught participants a rule about contingencies involving different symbols: one symbol was always followed by a manufactured object, and another by a natural object. Later, this contingency was altered to violate participants’ predictions, and retrieval rates were higher for items that violated prior predictions about contingency rules. Furthermore, in the study of Bein et al. (2021), where participants learned item–item associations, enhanced memory for pairs that violated the learned associations was observed.

These studies demonstrate that memory can benefit from both congruent and incongruent information depending on predictions and contextual factors. This underscores the importance of examining not only whether an event is remembered, but also how it is remembered, specifically, whether fine-grained perceptual details are encoded and can be retrieved. Recent research has begun to differentiate how the *congruency effect* affects recognition versus recall. For instance, Persaud et al. (2025) manipulated the congruency of object features, such as color (i.e., orange pumpkin vs. blue pumpkin), and examined their effects on memory. While recognition accuracy was similar for both congruent and incongruent object features, recall was significantly better for congruent features. This suggests that congruency may have qualitatively distinct effects depending on the memory process involved. Building on this perspective, rather than using individual objects with features that adhere to prior knowledge, the present experiment focuses on object–scene memory schemas to study how *contextual congruency* affects both recognition and retrieval of fine-grained perceptual details under congruent and incongruent conditions.

To empirically test these questions, we adopted the Mnemonic Similarity Task (MST) (Kirwan & Stark, 2007; Stark, Kirwan, & Stark, 2019; Stark & Stark, 2017), a paradigm designed to assess pattern separation of highly similar inputs and the extent to which they are encoded as distinct, non-overlapping memory representations. The MST consists of two phases: an incidental encoding phase, in which participants classify everyday object images (e.g., as “indoor” or “outdoor”), followed by a memory test phase. During the memory test phase, participants view a series of objects and must classify each as ”old” (exact repetitions of the objects presented at the encoding phase; *targets*), “similar” (images perceptually similar to those seen during the encoding phase, but not identical; *lures*), or “new” (new images not previously seen; *foils*). Critically, successful identification of *lures* as “similar” rather than “old” is taken as behavioral evidence of pattern separation, indicating that fine-grained perceptual details were encoded and retrieved to distinguish a *lure* from a *target*.

In the standard objects-based MST, *lures* can vary in multiple features (color, rotation, and other perceptual fine details), preventing participants from relying on a single feature strategy to discriminate items. As noted by Stark et al. (2019), if only one feature changed, such as color, participants could use a simple representation (e.g., a “red duck”) to reject a *lure* correctly. By varying multiple features, accurate classification requires a detailed retrieval of the original object. To assess memory performance in the MST, the Lure Discrimination Index (LDI) and the Corrected Recognition Memory (REC) score are used (Stark et al., 2019). The LDI captures a participant’s sensitivity to encode fine-grained perceptual details by assessing their ability to distinguish similar but not identical items (*lures*) from entirely new items (*foils*). It is computed as the proportion of “similar” responses to *lures* minus the proportion of “similar” responses to *foils*. In contrast, the REC score measures general encoding and recognition accuracy by calculating the difference between “old” responses to previously seen items (*targets*) and “old” responses to *foils* (i.e., hits minus false alarms). Although alternative measures of *lure* discrimination exist (e.g., comparing “similar” to “old” responses to *lures*), the LDI remains preferred due to its ability to account for potential response biases. Together, these indices allow researchers to evaluate not only how much information was encoded, but also how precisely the details of that information were encoded and can be retrieved.

Importantly, previous research by Bein et al. (2021) has used the MST to study the impact of prediction on memory, implementing a design in which participants learned fixed object pairs (e.g., a truck predicts a ball) and were then presented with either congruent or incongruent pairs during a violation phase. Memory was then tested using the MST, and participants showed enhanced memory for items that violated the learned predictions. Notably, in this study, the standard incidental encoding phase of the MST was replaced by an associative learning phase, allowing prediction effects to be examined. Although this design was effective in isolating prediction effects, it may not fully reflect how predictions are generated in everyday contexts. In natural settings, predictions are guided by preexisting knowledge and contextual schemas rather than newly learned associations.

Rather than relying on artificial learning phases, studies can draw on participants’ existing knowledge of object–scene associations to enhance contextual relevance (Bar, 2004). In the present study, as in many others using this rationale, naturalistic scenes were used to emphasize the role of realistic contexts in activating memory schemas during encoding (Võ 2021; Peelen, Berlot, & de Lange, 2024; Wischnewski & Peelen, 2021; Oliva & Torralba, 2007; Ortiz-Tudela et al., 2017; Ramey et al., 2022; Ramey et al., 2024). Within this framework, scene contexts are thought to shape predictions about which objects are likely to appear in a given environment, supporting the idea that predictions can be formed by computing on statistical regularities (Peelen et al., 2024; De Lange et al., 2018; Mudrik et al., 2010; Öhlschläger & Võ, 2017). Thus, the MST was modified for this purpose. During the encoding phase, object–scene pairings that were either congruent or incongruent were presented, and participants classified object images as “congruent” or “incongruent” with the context. In the subsequent memory test phase, participants classified single objects as “old,” “similar,” or “new.” This design allowed us to examine whether *contextual congruency* during the encoding phase enhances not only recognition of previously seen items (*targets*) as “old,” but also the ability to discriminate novel but similar items (*lures*) as “similar.” Although *lure* items presented at the memory test phase were not shown during the encoding phase, considering the nature of the MST task, successful discrimination may encourage participants to use a *recall-to-reject* strategy, in which the previously seen item (*target*) must be recalled and compared to reject the *lure* as ”old” and classify it as “similar” (Kirwan & Stark, 2007; Stark et al., 2019).

Despite advances in the field, it remains unclear whether contextual predictions based on prior knowledge enhance memory encoding and retrieval of fine-grained perceptual details for both congruent and incongruent information. Using the MST framework, the present study investigates how *contextual congruency*, grounded in memory schemas, influences both recognition memory and retrieval for fine-grained details, as measured by LDI and REC scores. Thus, the present study assessed whether recognition memory benefits emerge for congruent, incongruent, or both types of object–scene pairings, and to what extent detailed information is encoded in each case and can be retrieved.

## Method

### Participants

A total of 112 participants were initially recruited for this study through advertisements on social media that included a link to the experimental task. Participants were Mexican adults, primarily undergraduate students, with normal or corrected-to-normal vision. They confirmed consent to participate in the study before beginning the experiment.

Eight participants were excluded from the initial sample. Four participants were excluded because they reported conditions that affected their color perception, as this could have influenced their responses to images of objects where color was manipulated to distinguish similar lures in the item memory test. Although color perception was not essential for the task, this criterion was added to control potential confounding variables. Additionally, as in Bein et al. (2021), three participants were excluded due to poor performance in the encoding phase of the experiment, as they failed to achieve at least 80% accuracy in categorizing images of objects as congruent or incongruent with the context in which they were presented. This criterion ensured explicit encoding of *contextual congruency* with memory schemas. One participant was excluded due to low performance on the memory test during the memory test phase, with accuracy below 40% in both congruent and incongruent conditions. The final sample consisted of 104 participants (37 females, aged 18–31 years, $$M = 23.9$$, $$ SD = 2.28$$).

Given that the study was conducted online, we anticipated potential data loss due to technical issues or participant noncompliance. Therefore, recruitment exceeded the minimum sample size estimated for adequate statistical power, ensuring that the final sample would remain robust after applying exclusion criteria. Previous research supports this approach, showing that data collected online are as reliable as those obtained in laboratory settings, although larger samples are generally recommended to compensate for increased variability and potential data loss in online environments (Audrey & Shravan, 2024; Sauter, Stefani, & Mack, 2022).

### Materials

The stimuli consisted of 40 images of indoor scenes and 60 images of objects. For the first part of the experiment, the encoding phase (see Sect. “Encoding phase”), a total of 40 indoor scene images and 40 objects were selected from the ObScene database (Andrade, Cipriano, & Raposo, 2023). The ObScene database was chosen to address variability in previous findings from studies with different controls of semantic relationships (for a review of this issue, see Võ, 2021). The database contains real images of indoor and outdoor scenes paired with six object images, each with a semantic congruence score indicating the established associations between the object and the scene (Andrade et al., 2023). Semantic congruence was measured on a scale from 1 to 5, with higher scores indicating stronger associations, suggesting a higher likelihood of the object being present in that scene. Indoor scenes were selected based on whether at least one of the paired objects had a semantic congruence score above four or below two. Each of the 40 indoor scenes was paired with an object, 20 with a congruent object, and 20 with an incongruent object (i.e., with a high or low semantic congruence score).

The scene images were 1320 x 1080 pixels, covering most of the screen. Objects were displayed at the center of the scene within a white square (455 $\times$ 455 pixels). The objects were all scaled to match their size on the square by adjusting their dimensions so that the larger side (horizontal or vertical) fully occupied the 455 pixel length of the white square. The aspect ratio of each object was preserved to avoid distortion. While embedding objects directly into scenes (e.g., SCEGRAM database; Öhlschläger & Võ, 2017) can enhance realism, such approaches may introduce additional sources of variability, including object location and size. Placing objects on a neutral white background allowed for controlling these potential confounds, ensuring that attention remained focused on the target object in the scene and maintaining comparability across trials.

For the second part of the experiment, the memory test phase (see Sect. “Memory test phase”), 60 images of objects were used, classified as *targets*, *lures*, and *foils*, with 20 for each category. The *targets* were composed of previously presented items during the encoding phase (10 congruent and 10 incongruent), taken from the ObScene database (Andrade et al., 2023). The *lures* were images of objects that slightly differed in color or shape from the identical old objects. The similar lure items were selected from the internet (iStock, https://www.istockphoto.com), following the approach used by Bein et al. (2021). Lastly, the *foils* were new images of objects taken from the BOSS database (Brodeur, Dionne-Dostie, Montreuil, & Lepage, 2010). To maintain comparability and consistency across participants, all participants were exposed to the same set of stimuli, while the assignment of specific items used in the encoding phase to the memory test phase as *targets* or *lures* was randomized.

All stimuli were controlled to ensure they were indoor, non-animal, non-edible objects. The objects were all matched in size when displayed on the screen, and all were set against a white background. In addition, four outdoor scenes and four objects were used for the practice trials during the encoding phase, while three objects were used for the practice trials in the memory test phase.

**Fig. 1:**
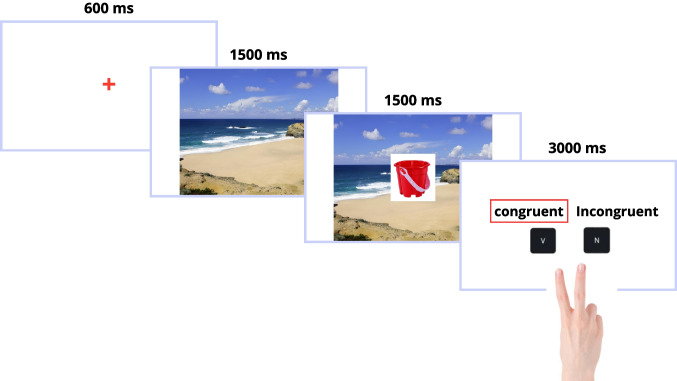
Encoding phase trial: Example of a congruent trial in which a scene context is presented after a semantically congruent object (i.e., high semantic congruence score). In this example, participants were required to classify the object as “congruent” with the scene to provide a correct response. The response keys, the *red rectangle* highlighting the correct response, and the hand are shown for illustrative purposes only. Note that while the labels in this illustration are in English, all stimuli and instructions were presented in Spanish during the actual experiment

### Procedure

The experiment was conducted online and lasted approximately 20 min with no breaks. The experiment was programmed using *jsPsych* version 7.3 (de Leeuw, 2014), conducted online on the GitHub platform, and utilized *DataPipe* (de Leeuw, 2024) to save data directly to the Open Science Framework (OSF) project web page.

At the beginning of the experiment, detailed instructions about the task were presented, emphasizing the importance of attention and quick responses. The MST was implemented (Kirwan, Jones, Miller, & Stark, 2006; Stark et al., 2019). In this experiment, the prediction learning phase typically used in the MST was excluded, as participants relied on object–scene prior knowledge. The experiment was conducted in two phases: the encoding phase (see Sect. “Encoding phase”) and the memory test phase (see Sect. “Memory test phase”). Before starting each phase, participants completed practice trials.

There were four practice trials for the encoding phase (two for the congruent condition and two for the incongruent condition) and three for the memory test phase (one for each type of stimulus: *targets*, *foils*, and *lures*), during which they received feedback and were informed that they would not receive feedback during the actual test. They were additionally asked to respond as quickly as possible while still being accurate.

#### Encoding phase

During the encoding phase, a total of 40 trials were conducted, presenting 40 images of indoor scenes one at a time, each accompanied by a congruent or incongruent object (for an example of a congruent trial, see Fig. 1). In each trial, an image of an indoor scene was presented on the screen for 1.5 s. Then, an object appeared in the center of the scene (which could be congruent or incongruent with the background scene) for another 1.5 s. After this, the words “Congruent” and “Incongruent” were displayed on the left and right sides of a white screen. The position of these words was counterbalanced across trials. The participants had to indicate whether the object was congruent or incongruent with the background scene. They used the keyboard to respond by pressing the “V” key for the option on the left side of the screen or the “N” key for the option on the right side. Participants had a maximum of 3 s to respond. Following each trial, a 600-ms fixation cross was displayed at the center of the screen as an interstimulus interval (ISI). The order of the scenes was randomized for each participant.

**Fig. 2:**
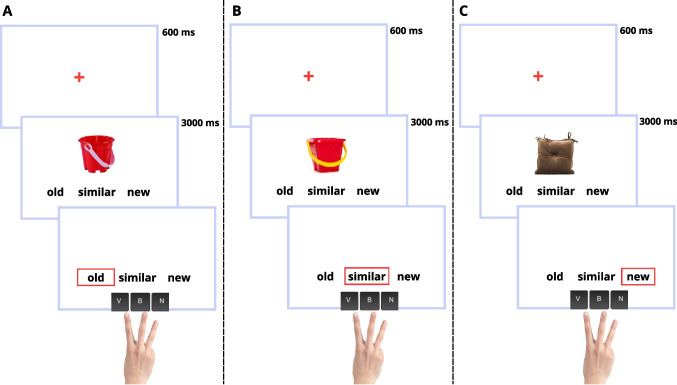
Examples of the three types of items presented during the memory test, based on the object shown in the example of the encoding phase (see Fig. 1). (**A**) *Target* – identical to an object encountered during the encoding phase, requiring a response of ”old” to be correct; (**B**) *Lure* – perceptually similar but not identical to a *target*, requiring a response of ”similar”; (**C**) *Foil* – not previously encountered during the encoding phase, requiring a response of ”new”. The response keys, the hand, and the *red rectangle* highlighting the correct response are included for illustrative purposes only

#### Memory test phase

A total of 60 trials were conducted, presenting one item that could correspond to a *target*, *lure*, or *foil*, which had to be classified by participants as ”old”, ”similar”, or ”new”, at each trial (for an example of each type of item and their correct classification, see Fig. 2). Twenty objects were presented for each type of item. Participants were required to classify each item as:

- “Old” if they considered the presented stimulus identical to one they had observed in the encoding phase (*target*).
- “Similar” if they thought the stimulus was similar but not identical to one they observed in the encoding phase (*lure*).
- “New” if they believed the stimulus had never been presented before in the experiment (*foil*).

Below each object, the response options (“Old” - “Similar” - “New”) were displayed as a guide for participants. The order of options presented on the screen was counterbalanced among participants. The response keys were as follows: “V” for the left response, “B” for the middle response, and “N” for the response on the right. The object was removed from the screen after 3 s of presentation. The response options remained visible until the participant made their response. There was no time limit for the participants’ responses, although they were instructed to respond as quickly as possible while maintaining accuracy. A 600-ms fixation cross was displayed at the center of the screen as an interstimulus interval (ISI). Participants had a brief practice session, consisting of one trial for each type of object (old, similar, and new), during which they received feedback. They were informed that no feedback would be provided during the actual test. The order of presentation of all objects was counterbalanced across participants to avoid systematic order effects.

## Results

For the analysis of all results, the *RStudio* software version 2024.12.0+467 was used (Posit team, 2024). Inspired by the mnemonic similarity task analysis of Stark and Stark (2017) and Bein et al. (2021), a similar analysis was conducted and adapted to the conditions of this experiment.

**Table 1:** Frequencies (*n*) and proportions of object classification responses (“New” - “Old” - “Similar”) across item types

Type	*n*	“New”	*n*	“Old”	*n*	“Similar”	*N*
*Foil*	1813	**0.872**	38	0.0183	229	0.110	2080
$T_C$	25	0.0240	924	**0.888**	91	0.0875	1040
$T_I$	41	0.0394	857	**0.824**	142	0.137	1040
$L_C$	46	0.0442	305	0.293	689	**0.662**	1040
$L_I$	93	0.0894	264	0.254	683	**0.657**	1040

$T_C$ = *Target* items that were Congruent with the scenes; $T_I$ = *Target* items that were Incongruent; $L_C$ = *Lure* items that were Congruent; $L_I$ = *Lure* items that were Incongruent. **N** refers to the total number of responses for each item type. The proportion of correct responses is highlighted in bold

**Table 2:** Descriptive statistics of memory test scores

Memory test score	M	SD
$LDI_C$	0.552	0.247
$LDI_I$	0.547	0.216
$REC_C$	0.870	0.115
$REC_I$	0.806	0.148

M = Mean; SD = Standard Deviation. $LDI_C$ and $REC_C$ refer to congruent conditions; $LDI_I$ and $REC_I$ refer to incongruent conditions

The full distribution of responses by the participants classifying objects as “new”, “similar”, and “old” for each item type (Foil, Target, Lure) is presented in Table 1. These descriptive statistics provide a direct characterization of participants’ discrimination patterns prior to the computation of LDI and REC scores. *Foil* items were predominantly classified correctly as “new”, indicating low false-alarm rates. *Target* items were most frequently and accurately classified as “old”, with slightly reduced accuracy in the incongruent condition. *Lure* items were most often classified as “similar”, but a notable proportion of responses, approximately one-third, were misclassified as “old”, reflecting the difficulty of discriminating “similar” from “old” objects.

### Contextual congruency and memory

Following the analysis of the MST (Bein et al., 2021; Kirwan et al., 2012, 2006; Stark et al., 2019; Stark & Stark, 2017), the LDI and REC scores were calculated for each participant under the congruent and incongruent conditions (Table 2). These memory tests scores are essential for the present research, as they provide a dissociation between general recognition accuracy (REC) and fine-grained memory discrimination (LDI), as well as they counter potential response biases by considering both the total of “similar” or “old” classification of objects and their respective correct and incorrect responses, as well as the total number of “new” responses. LDI and REC scores were calculated as follows:

$$\begin{aligned} LDI = \left( \frac{{\text {correct similar}}}{{\text {total similar}}}\right) - \left( \frac{{\text {incorrect similar}}}{{\text {total new}}}\right) \end{aligned}$$

$$\begin{aligned} REC = \left( \frac{{\text {correct old}}}{{\text {total old}}}\right) - \left( \frac{{\text {incorrect old}}}{{\text {total new}}}\right) \end{aligned}$$

A generalized linear mixed-effects model (GLMM) with a beta distribution and a logit link function was fitted to the memory test scores to examine the effects of *Type* of memory test (*REC*, *LDI*) and *Condition* (Congruent, Incongruent). A random intercept for each participant (*ID*) was included to account for within-subject variability due to repeated measures. Categorical predictors for *Type* of memory test and *Condition* had *LDI* and *Congruent* as reference levels, respectively. Following standard practice for beta regression, memory test scores were adjusted ($\epsilon = 10^{-4}$) to fall within the open interval (0, 1). Model fitting was conducted using the glmmTMB package in R (Brooks et al., 2017). A likelihood ratio test indicated that the model including the interaction term *Type*$\times$*Condition* provided a significantly better fit than the additive model, $\chi ^2(1) = 14.03$, $p < 0.001$ (Interaction model: AIC = $-546.05$; Additive model: AIC = $-534.02$). Additionally, the Bayes Factor (BF = 54.44) provided strong evidence in favor of the interaction model over the additive model. Thus, all subsequent analyses and interpretations are based on the interaction model, formally specified as:

$$\begin{aligned} \texttt {Scores} \sim \texttt {\textit{Type}.memory.test} * \texttt {\textit{Condition}} + (1 \mid \texttt {ID}) \end{aligned}$$

Model assumptions were assessed using the *performance* package in R (Lüdecke, Ben-Shachar, Patil, Waggoner, & Makowski, 2021). Diagnostic checks showed no substantial violations: residuals were approximately uniform, variance appeared homoscedastic, and no problematic multicollinearity was detected (all VIFs $< 5$). Model performance indices indicated strong explanatory power, with a conditional $R^2 = 0.947$, marginal $R^2 = 0.629$, and RMSE = 0.138. The intraclass correlation coefficient (ICC = 0.858) indicated that 85.8% of the total variance in memory scores was due to between-participant differences.

**Fig. 3:**
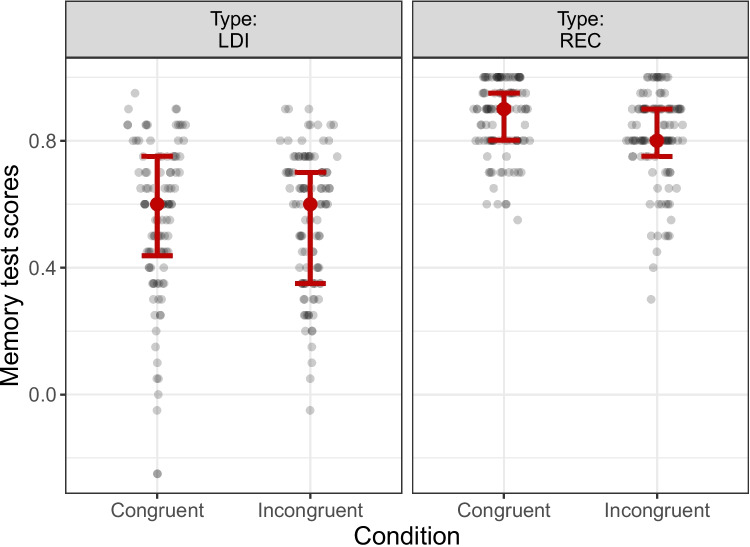
Distribution of memory test scores (LDI and REC) across congruent and incongruent conditions, plotted using the *flexplot* package in R (Fife, 2022). *Gray dots* represent individual observations. *Red dots* indicate group means with 95% confidence intervals (*red bars*)

**Table 3:** Pairwise comparisons of memory test scores (odds ratios) by test type (LDI, REC) and condition (C = Congruent, I = Incongruent)

Contrast	OR	SE	95% CI	*z*	*p*
LDI$_C$ vs. LDI$_I$	0.946	0.109	[0.70, 1.28]	$-0.48$	$=0.997$
REC$_C$ vs. REC$_I$	1.863	0.258	[1.29, 2.68]	4.49	$<0.001$
LDI$_C$ vs. REC$_C$	0.123	0.016	[0.08, 0.17]	$-15.72$	$<0.001$
LDI$_I$ vs. REC$_I$	0.241	0.031	[0.17, 0.33]	$-11.11$	$<0.001$
LDI$_C$ vs. REC$_I$	0.228	0.029	[0.16, 0.31]	$-11.80$	$<0.001$
REC$_C$ vs. LDI$_I$	7.723	1.060	[5.38, 11.07]	14.93	$<0.001$

OR = odds ratio; SE = standard error; CI = confidence interval. Odds ratios are back-transformed from the log-odds scale. For each contrast, an OR > 1 indicates higher odds for the first (left) condition

The GLMM revealed a significant main effect of *Type* of memory test, with significantly higher scores for *REC* compared to *LDI* ($\beta = 2.10$, SE = 0.13, $z = 15.72$, $p < 0.001$, 95% CI [1.84, 2.36]). To facilitate interpretation, the coefficient ($\beta$) was exponentiated to obtain an odds ratio (OR): OR = $e^{2.10} = 8.17$, indicating 8.17 times greater odds of higher REC scores representing a substantial difference in memory performance, confirmed by a large standardized effect ($\beta _{\text {std}} = 1.05$, 95% CI [0.92, 1.18]). There was no evidence of a significant main effect of *Condition* ($\beta = 0.055$, SE = 0.11, $z = 0.48$, $p = 0.632$, 95% CI [$-0.17$, 0.28]; $\beta _{\text {std}} = 0.03$, 95% CI [–0.09, 0.14]). Crucially, there was a significant *Type* of memory test $\times$ *Condition* interaction ($\beta = -0.68$, SE = 0.18, $z = -3.78$, $p < 0.001$, 95% CI [$-1.03$, $-0.33$]), indicating differential congruency effects across memory test types (see Fig. 3). The interaction (OR = $e^{-0.68} = 0.51$; $\beta _{\text {std}} = -0.29$, 95% CI [–0.45, –0.14]) shows that *contextual congruency* effects in LDI are approximately half of that observed for REC scores, suggesting that performance on recognition is more sensitive to *contextual congruency*. The random intercept variance was 0.4187, with a standard deviation of 0.647 logit units (95% CI [0.530, 0.791]).

Pairwise contrasts (Sidak-adjusted, see Table 3) were conducted using the *emmeans* package (Russell et al., 2018) to interpret the significant *Type* of memory test $\times$ *Condition* interaction. These contrasts confirm the interaction pattern, showing that for *REC*, the congruent condition scores were significantly higher than in the incongruent condition (OR = 1.86, 95% CI [1.29, 2.68], $p < 0.001$), indicating that the odds of higher performance are 1.86 times greater in the congruent compared to the incongruent condition. For the memory test *LDI*, there was no evidence of a significant difference between congruent and incongruent conditions (OR = 0.95, 95% CI [0.70, 1.28], $p = 0.997$), indicating that *contextual congruency* does not enhance the discrimination of *lures*, which likely requires memory retrieval for fine-grained details. All other pairwise comparisons between *LDI* and *REC* memory test scores were statistically significant ($p < 0.001$), consistently showing that *REC* memory test scores were higher than *LDI* scores across both congruent and incongruent conditions.

### Rate of correct scores

In addition to analyzing the LDI and REC memory test scores, the response latencies of the participants were examined as a measure of cognitive performance. This analysis focused on the memory test phase, where participants had to decide if an object was “new”, “old”, or “similar”. For the present analysis, only responses for *target* and *lure* items were included, as the experimental hypotheses focused on the effects of *contextual congruency* in memory performance.

**Table 4:** Descriptive statistics for error rates (percentages) and response latencies (milliseconds) across item types and conditions

Condition	Error Rate (%)		Response Latency (ms)	
	M	SD	M	SD
$T_C$	11.2	11.7	1465	319
$T_I$	17.6	14.8	1545	373
$L_C$	33.8	19.8	1647	355
$L_I$	34.3	17.9	1626	390

**Fig. 4:**
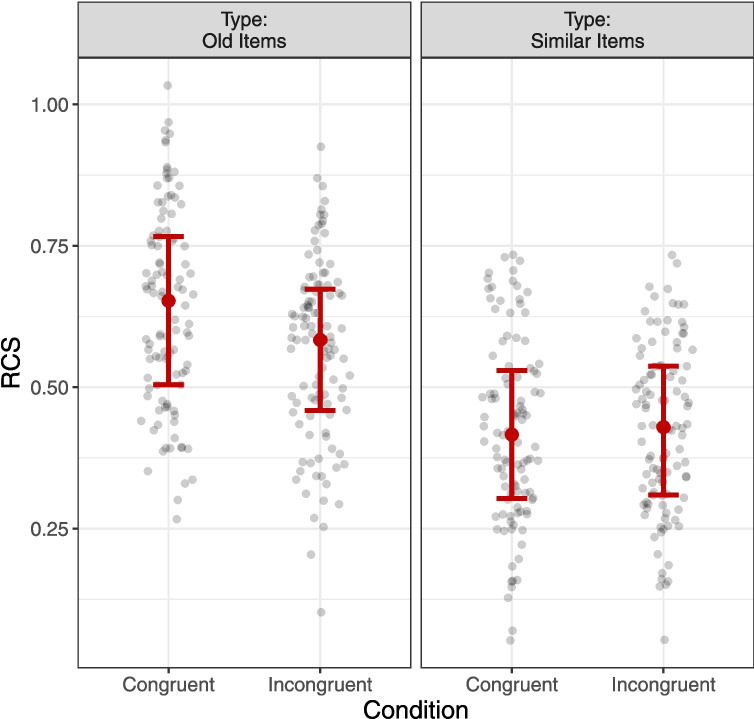
Each panel displays the RCS (correct responses per second) for *target* (labeled as Old items) and *lure* (labeled as Similar items), separated by Condition. Higher RCS values reflect processing efficiency (faster and more accurate responses). *Gray dots* represent individual observations. *Red dots* indicate group means with 95% confidence intervals

Response latencies were analyzed considering the trade-off between speed and accuracy (Vandierendonck, 2021), as a high error rate may reflect the difficulty of the task or a trade-off between speed and accuracy related to participants’ strategies and experimental demands. To capture both response speed and accuracy, a composite performance measure called the Rate Correct Score (RCS) was calculated, incorporating latency and error rate, following the rationale proposed by Woltz and Was (2006). Notably, the RCS reflects the number of correct responses per unit time and serves as an index of processing efficiency, adjusted for errors. All RCS were calculated as follows:

$$\begin{aligned} \text {RCS}_{ij} = \frac{\sum \text {Correct}_{ij}}{\sum \text {RL}_{ij} / 1000} \end{aligned}$$

where $\text {RCS}_{ij}$ denotes the Rate Correct Score for participant *i* in *Condition* (congruent, incongruent) and specific item *Type* (*target*, *lure*) *j*, $\text {Correct}_{ij}$ is the sum of the total number of correct responses in that condition (correct stimulus classification as “old”, or “similar”), and $\text {RL}_{ij}$ is the sum of the total response latencies in milliseconds (for both correct and incorrect responses). The denominator is divided by 1000 to express RCS in units of correct responses per second. Descriptive statistics for mean latencies and error rates from which all RCS were calculated are provided in Table 4.

A linear mixed-effects model was fitted to predict Rate Correct Scores (RCS) as a function of *Type* of item (*target* vs. *lure*) and *Condition* (congruent vs. incongruent), along with their interaction, with random intercepts for participants (ID). The *lme4* package in R was used (Bates et al., 2015). Categorical predictors for *Type* of item and *Condition* had *Target* and Congruent as reference levels, respectively. To evaluate the contribution of the interaction between *Type* of item and *Condition* on response efficiency, two linear mixed-effects models were compared, one including only the main effects, and another including their interaction term. The comparison was performed using a likelihood ratio test with maximum likelihood (ML) estimation. Results indicated that the model including the interaction provided a significantly better fit to the data, $\chi ^2(1) = 8.78$, $p = 0.003$, with a lower AIC value (AIC = -377.71) compared to the additive model (AIC = -370.93). Additionally, the Bayes factor (BF = 3.98) provided moderate evidence in favor of the additive model. However, the interaction was retained given the strong statistical evidence from the likelihood ratio test and its theoretical relevance. All subsequent analyses and interpretations are based on the interaction model, specified as:

$$\begin{aligned} \texttt {RCS} \sim \texttt {\textit{Type}.item} * \texttt {\textit{Condition}} + (1 \mid \texttt {ID}) \end{aligned}$$

The interaction model was estimated using restricted maximum likelihood (REML), and *t*-values were computed using Satterthwaite’s approximation for degrees of freedom. Categorical predictors had *old* and *congruent* as reference levels. Confidence Intervals were computed using the Wald approximation. Model assumptions were evaluated using diagnostic plots, which indicated acceptable linearity, homoscedasticity, normality of residuals, absence of high-leverage observations, and no problematic collinearity (all VIFs $< 5$). Model performance indices demonstrated good explanatory power, with a conditional $R^2$ of 0.492, a marginal $R^2$ of 0.246, and an RMSE of 0.121. The inclusion of a random intercept for participants accounted for individual differences (ICC = 0.326), indicating that 32.6% of the total variance was attributable to between-participant differences.

The model revealed a significant main effect of *type* of item, with lower RCS for *lure* compared to *target* items ($\beta = -0.216$, SE = 0.018, 95% CI [$-0.25$, $-0.18$], $t(309) = -11.70$, $p < 0.001$; $\beta _{\text {std}} = -0.58$, 95% CI [$-0.68$, $-0.48$]), indicating reduced processing efficiency for *lure* items. A significant main effect of *Condition* showed lower RCS for incongruent compared to congruent conditions ($\beta = -0.075$, SE = 0.018, 95% CI [$-0.11$, $-0.04$], $t(309) = -4.09$, $p <.001$; $\beta _{\text {std}} = -0.20$, 95% CI [$-0.30$, $-0.11$]). Crucially, a significant *Type* of item $\times$ *Condition* interaction emerged ($\beta = 0.077$, SE = 0.026, 95% CI [0.03, 0.13], $t(309) = 2.97$, $p = 0.003$; $\beta _{\text {std}} = 0.18$, 95% CI [0.06, 0.30]), indicating differential *contextual congruency* effects on processing efficiency across item types (see Fig. 4). The random intercept variance was 0.008, with a standard deviation of 0.093, and the residual variance was estimated at 0.017.

Pairwise comparisons of estimated marginal means (Sidak-corrected) revealed that *contextual congruency* effects differed by *Type* of item (see Table 5). Performance for *target* items that were presented on congruent scenes ($T_C$) was significantly higher than for those presented on incongruent scenes ($T_I$), indicating a processing efficiency effect of congruency for items previously encountered. In contrast, no difference was observed between *lure* items that were presented on congruent scenes ($L_C$) and those presented on incongruent scenes ($L_I$), suggesting that congruency did not affect processing efficiency for *lure* items. All remaining comparisons were statistically significant.

**Table 5:** Pairwise comparisons of RCS by type of item and condition

Contrast	Estimate	SE	95% CI	*t*	*p*
$T_C$ vs. $T_I$	0.075	0.018	[0.02, 0.12]	4.09	$<0.001$
$L_C$ vs. $L_I$	-0.002	0.018	[-0.05, 0.04]	-0.11	$= 1.00$
$T_C$ vs. $L_C$	0.216	0.018	[0.16, 0.26]	11.70	$<0.001$
$T_C$ vs. $L_I$	0.214	0.018	[0.16, 0.26]	11.58	$<0.001$
$T_I$ vs. $L_I$	0.138	0.018	[0.08, 0.18]	7.50	$<0.001$
$L_C$ vs. $T_I$	-0.140	0.018	[-0.18, -0.09]	-7.61	$<0.001$

## Discussion

The conflicting perspectives on whether memory is enhanced for events that match our predictions or for those that violate them pose a theoretical challenge for the current literature on episodic memory. The present study aimed to investigate how *contextual congruency* based on memory schemas influences both recognition and retrieval for fine-grained details, a less explored dimension of memory performance. To address this, a paradigm that relies on prior knowledge of the participants was used to generate predictions based on memory schemas of context and systematically manipulate object likelihood to create congruent and incongruent object–scene pairings, without the need for artificial learning phases. Memory was assessed using the Lure Discrimination Index (LDI) and Corrected Recognition (REC) (Stark et al., 2019; Stark & Stark, 2017). These two complementary measures capture not only how much information is remembered and can be recognized, but also the level of perceptual detail retained in memory. Results indicate that contextual congruency enhances recognition accuracy and increases the processing efficiency of information, as reflected in the REC scores and the Rate of Correct Scores (RCS), respectively. In contrast, no congruency advantage was observed for the retrieval of fine-grained perceptual details, as indicated by the LDI scores, which were comparable across congruent and incongruent conditions. Thus, while schema-consistent information facilitated recognition and processing efficiency, it did not facilitate the encoding and retrieval of perceptual details.

The present findings are consistent with previous research, which has shown performance differences between LDI and REC memory test scores (Kirwan et al., 2012; Stark et al., 2019; Stark & Stark, 2017). In general, REC scores were significantly higher than LDI scores, supporting the notion that recognizing previously encountered items, *targets*, as “old” is less cognitively demanding than discriminating *lures* as “similar”. Discriminating a *lure* requires not only recognizing its similarity to a previously encoded *target* but also retrieving fine-grained perceptual details to determine that the two items are not identical. The observed pattern in the present results, in which *contextual congruency* enhanced recognition of *target* items but did not significantly benefit the discrimination of *lures*, suggests that *contextual congruency* primarily supports recognition processes based on familiarity, rather than detailed encoding and retrieval. Additionally, the REC scores were significantly higher in the congruent condition in comparison to the incongruent condition, indicating a *contextual congruency* effect in recognition performance when object–scene pairs were aligned with predictions based on memory schemas. In contrast, LDI scores did not differ significantly between congruent and incongruent conditions, suggesting that the encoding of fine-grained perceptual details was not modulated by *contextual congruency*.

The processing efficiency analysis using the Rate of Correct Scores (RCS) provides converging evidence for these findings. The significant interaction between item type and condition revealed that *contextual congruency* selectively enhanced processing efficiency (faster and more accurate responses) for *target* items but not for *lure* items. This pattern suggests that predictions based on memory schemas facilitate the encoding and retrieval of general item information while having minimal impact on fine-grained perceptual details. This interpretation aligns with theoretical frameworks proposing that schema-consistent information is more easily integrated into preexisting memory networks, promoting rapid consolidation and efficient retrieval (Alonso et al., 2020; Audrain & McAndrews, 2022; van Kesteren et al., 2012). By contrast, the specific perceptual details necessary for discriminating *lure* items do not benefit as clearly from *contextual congruency* and may rely on different encoding mechanisms. These differential effects between recognition and retrieval of perceptual details offer important insights into how predictions influence distinct aspects of memory encoding.

It is worth noting that other paradigms have shown different patterns. For example, in drawing-based free recall tasks, incongruent objects are remembered more vividly than congruent ones (Bainbridge, Hall, & Baker, 2019). However, this benefit for the incongruent object often comes at the expense of memory for other scene elements. These contrasting findings highlight the importance of comparing recognition-based and free recall paradigms to gain a deeper understanding of how prediction and *contextual congruency* influence memory encoding and retrieval. The differences across paradigms may reflect distinct memory processes: recognition tasks may preferentially engage familiarity-based processing that benefits from schema consistency. At the same time, free recall may recruit more elaborate encoding processes that are enhanced by distinctiveness. These findings, along with those of the present study, suggest the need to test these ideas collectively, evaluating whether memory encoding is enhanced for general, highly congruent, or incongruent information over fine-grained details. Additionally, studying the differences between experimental paradigms that require recognition or free recall of stimuli is still necessary to unravel specific encoding and retrieval mechanisms in memory.

The *congruency effect* observed here belongs to a broader set of memory phenomena that emphasize the influence of prior knowledge on new memory formation (Bein et al., 2015; Brod & Shing, 2022; Craik & Tulving, 1975; Schulman, 1974). In line with the results, it has been found that object–scene pairs that align with semantic knowledge are typically processed, integrated, and remembered more easily, as experiences are encoded in relation to prior knowledge (Audrain & McAndrews, 2022; Ortiz-Tudela et al., 2017; van Kesteren et al., 2013). Consistent with this, studies on object features (e.g., color, shape) show that predicted congruent information tends to be better remembered, whereas the benefits for incongruent features are less clear (Persaud et al., 2025). Together, these findings suggest that *contextual congruency* facilitates memory for object-level perceptual features. Understanding how different types of information (e.g., semantic, perceptual, episodic) interact with *contextual congruency* remains an open question. Additionally, it is still necessary to know in which cases incongruent information is prioritized over congruent information, and to what extent fine-grained perceptual details will be integrated into existing memory schemas.

A critical theoretical question concerns whether prediction effects require explicit preactivation of specific representations (Ortiz-Tudela et al., 2023) or whether predictions that are based on prior knowledge of statistical regularities of the environment suffice. In the present findings, the fact that a significantly higher recognition performance was observed on the congruent conditions in comparison to the incongruent condition, and that no fine-grained perceptual details were encoded and retrieved in both conditions, does not imply that contextual predictions and prediction error were absent or irrelevant to memory processing and encoding. Rather, the significant processing efficiency differences between congruent and incongruent items demonstrate that these conditions engaged distinct cognitive operations. The present findings support the idea that *contextual congruency*, based on probabilistic estimation of incoming sensory information derived from learned regularities, influences recognition and processing efficiency without the need for an explicit prediction. This is consistent with evidence showing that N300 component amplitude increases for statistically irregular scenes, even when no specific scene category is predicted in advance, suggesting that congruency effects can arise from implicit template matching processes rooted in prior experience (Kumar et al., 2021). However, continued investigation will be essential to address this issue.

Predictive coding theory proposes that the brain continuously generates probabilistic predictions based on prior knowledge, with prediction precision (i.e., inverse of variance) modulating prediction error weighting (Clark, 2013; Friston, 2018; Friston & Kiebel, 2009). From a Bayesian perspective on prediction-error-driven learning, which is closely related to predictive coding models of memory, prediction error reflects the divergence between prior and likelihood probability distributions about sensory information (van Kesteren et al., 2012). Critically, maximal novelty does not necessarily entail maximal prediction error: when both prior and likelihood are imprecise (novel object in novel context), prediction error remains low. In contrast, when a familiar but unexpected object appears in a familiar context (e.g., a penguin on a busy, noisy, familiar street), both distributions are precise but divergent, generating high prediction error. Memory performance, however, depends strongly on the retrieval cue (van Kesteren et al., 2012). Thus, the consequences of prediction error for memory cannot be fully understood without considering how information is later retrieved, since different cues may emphasize distinct aspects of the encoded episode.

In line with this cue-dependence, in the present study, memory was tested using objects as retrieval cues: participants viewed individual objects and judged whether they were “old”, “similar”, or “new.” Under these conditions, congruent items showed enhanced recognition and processing efficiency, likely because schema-consistent objects benefit from prior-likelihood overlap during encoding. However, if contexts had been used as retrieval cues instead (e.g., presenting scenes and asking participants to retrieve which objects appeared), incongruent items might have shown a recognition advantage. Moreover, according to van Kesteren’s SLIMM framework (Schema-Linked Interactions between Medial prefrontal and Medial temporal regions) (van Kesteren et al., 2012), incongruent experiences trigger structures within the medial temporal lobe (MTL), such as the hippocampus, to mediate instance encoding that binds together all elements of the experience, including incidental episodic details. Thus, when the context is used as a retrieval cue, participants may retrieve fine-grained perceptual details of both the distinctive context (e.g., specific features of the street where the penguin appeared) and the unexpected object more accurately than in congruent pairings, in which the medial prefrontal cortex (mPFC) tends to integrate more generalized memories and suppress schema-irrelevant details. One limitation of the present work is that a context-cued paradigm was not implemented to compare the results with the object-cue paradigm that was used and to directly test whether context-based retrieval would have shifted the balance in favor of incongruent pairings, as predicted by SLIMM. Future research employing context-cued retrieval paradigms could test whether the pattern of results found here about the effects of congruency reverses, with incongruent pairings showing enhanced memory for both the context and the incongruent object.

An alternative, yet complementary, explanation is that while incongruent items may have generated high prediction error due to their low semantic likelihood, this error was not behaviorally relevant for the task at hand. In line with predictive coding models, prediction errors enhance memory primarily when they are task-relevant or demand updating of internal models. In the present experiment, participants were not required to encode contextual associations. Instead, they were asked to decide if the object presented in the scene was congruent or incongruent. Thus, incongruent items, although surprising, may not have triggered sufficient encoding resources for detailed memory consolidation. This interpretation aligns with findings showing that intermediate or even high prediction errors do not enhance memory (Ortiz-Tudela, Milliken, Jiménez, & Lupiáñez, 2018; Quent et al., 2021; van Kesteren et al., 2012). Future studies should systematically vary the task relevance of prediction error, in addition to its magnitude and precision, to better understand when incongruent information is encoded or suppressed. Such investigations could clarify under which conditions prediction errors are incorporated into existing schemas or stored as distinctive episodic instances.

These considerations resonate with mounting evidence for non-linear relationships between prediction and memory. Whereas some studies report a U-shaped function—showing enhanced memory for both highly expected and highly unexpected items (Frank et al., 2018; Greve, Cooper, Tibon, & Henson, 2019; Quent et al., 2022; van Kesteren et al., 2012), others have found an inverted U-shape, particularly under conditions involving explicit predictions (Ortiz-Tudela et al., 2023). The present study tested only items at the extremes of the congruency spectrum according to the ObScene database (high congruence and low congruence). A limitation of this work is that it does not allow testing U-shaped or inverted U-shaped functions relating prediction error magnitude to memory performance. Thus, to address these seemingly contradictory findings regarding the U-shape or inverted, future research could systematically manipulate semantic congruency using validated ratings from Andrade et al. (2023) to induce varying degrees of prediction error, together with manipulations of context familiarity (precision) and prediction explicitness. Such a design could clarify whether memory benefits follow a U-shaped, inverted U-shaped, or as predicted by the SLIMM framework.

## Conclusion

The present study investigated how *contextual congruency* based on memory schemas influences both recognition memory and the encoding of fine-grained perceptual details. This research originated from the observation that ongoing debates about whether congruent or incongruent information leads to better episodic memory could benefit from paradigms that rely on memory schemas of context, as well as measures of performance on recognition and the retrieval of fine-grained perceptual details, a less explored dimension of memory performance. Using an adapted Mnemonic Similarity Task, memory was assessed by presenting congruent and incongruent object–scene pairings, and then evaluating its effects on object recognition, retrieval of fine-grained perceptual details, and processing efficiency. Results revealed that *contextual congruency* selectively enhanced recognition accuracy and processing efficiency for previously encountered objects while having no measurable effect on retrieving and discriminating fine-grained perceptual details. These differential effects demonstrate that object-cued recognition benefits from schema-based predictions generated through prior knowledge, whereas detailed perceptual encoding appears less influenced by information that aligns or violates predictions. These findings highlight that the relationship between predictions and episodic memory remains incompletely characterized. Here, complementary approaches that warrant further investigation are suggested: (a) the use of context-cued retrieval paradigms, (b) high, medium, and low likelihood of object–scene combinations to manipulate prediction error magnitude, together with (c) the familiarity of the contexts presented in the scene, and (d) systematically comparing the results when using implicit probabilistic predictions and explicit predictions. Ultimately, these investigations will reveal that the question is not whether memory favors congruent or incongruent information, but rather when and how each serves the adaptive goals of building stable knowledge while remaining sensitive to updating this knowledge when required.

### Supplementary information

Not applicable.

## Data Availability

The datasets generated and/or analyzed during the current study are available in the OSF repository (https://osf.io/jq435/?view_only=224385e665de4cd0b89fa0bbc5a3081c). None of the experiments was preregistered.

## Materials Availability

All stimuli used in the current study are available in the OSF repository.

## Code Availability

The code of the experiment is available in the OSF repository.
